# Illuminating Spatial and Temporal Organization of Protein Interaction Networks by Mass Spectrometry-Based Proteomics

**DOI:** 10.3389/fgene.2015.00344

**Published:** 2015-12-01

**Authors:** Jiwen Yang, Sebastian A. Wagner, Petra Beli

**Affiliations:** ^1^Institute of Molecular Biology, Mainz, Germany; ^2^Department of Medicine, Hematology and Oncology, Goethe University, Frankfurt, Germany

**Keywords:** mass spectrometry-based proteomics, protein–protein interactions, transient interactions, spatial interactions

## Abstract

Protein–protein interactions are at the core of all cellular functions and dynamic alterations in protein interactions regulate cellular signaling. In the last decade, mass spectrometry (MS)-based proteomics has delivered unprecedented insights into human protein interaction networks. Affinity purification-MS (AP-MS) has been extensively employed for focused and high-throughput studies of steady state protein–protein interactions. Future challenges remain in mapping transient protein interactions after cellular perturbations as well as in resolving the spatial organization of protein interaction networks. AP-MS can be combined with quantitative proteomics approaches to determine the relative abundance of purified proteins in different conditions, thereby enabling the identification of transient protein interactions. In addition to affinity purification, methods based on protein co-fractionation have been combined with quantitative MS to map transient protein interactions during cellular signaling. More recently, approaches based on proximity tagging that preserve the spatial dimension of protein interaction networks have been introduced. Here, we provide an overview of MS-based methods for analyzing protein–protein interactions with a focus on approaches that aim to dissect the temporal and spatial aspects of protein interaction networks.

## Protein Interactions are Defined by Temporal and Spatial Constraints

**FIGURE 1:**
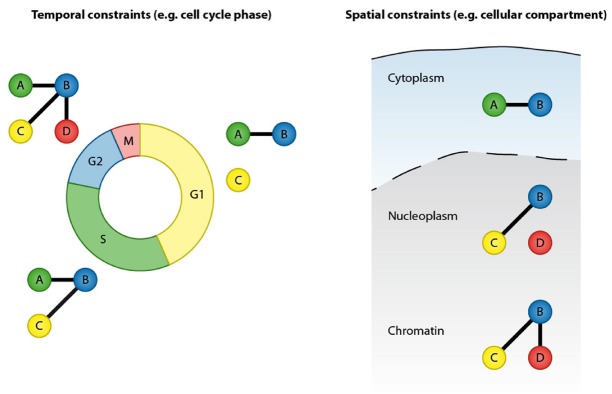
**Protein–protein interactions are defined by temporal and spatial constraints.** Many protein interactions are transient and occur only at specific time points, for instance in a particular cell cycle stage. These transient interactions can be mediated by posttranslational modification or by dynamic changes in expression of the binding partners. In addition to temporal constrains, protein interactions are spatially restricted by cellular compartments.

Protein–protein interactions are at the core of all cellular functions and dynamic alterations in protein interactions regulate cellular signaling (Scott and Pawson, 2009). Accurate and comprehensive mapping of protein–protein interaction networks is essential for understanding the regulatory mechanisms of cellular processes and signaling pathways as well as for identifying perturbed cellular signaling underlying human diseases. Proteins can form stable interactions and function as part of permanent protein assemblies, however a large proportion of protein–protein interactions are defined by temporal and spatial constraints. Protein–protein interactions can be dynamically altered in response to the intrinsic and extrinsic stimuli (Perkins et al., 2010). Transient protein interactions are frequently induced by posttranslational modifications (PTMs) and, depending on their cellular function, have a range of affinities and lifetimes (Nooren and Thornton, 2003; Seet et al., 2006). Prominent examples include the recruitment of DNA repair factors to sites of DNA lesions, cell cycle-regulated interactions and the formation of receptor signaling complexes after growth factor stimulation. Furthermore, protein–protein interactions are restricted by cellular compartments and can be regulated by protein re-localization to specific cellular structures or organelles. The transient nature and spatial organization are therefore important features that need to be considered when analyzing protein–protein interaction networks (Figure 1).

## Mass Spectrometry-Based Proteomics for Analysis of Protein–Protein Interactions

Mass spectrometry (MS)-based proteomics has become an indispensable tool in modern molecular and cell biology research (Larance and Lamond, 2015). In shotgun or bottom up proteomics approaches, proteins are extracted from cells or tissues and digested into peptides using specific proteases (Aebersold and Mann, 2003). The resulting peptides are separated according to hydrophobicity using high-pressure liquid chromatography and identified by tandem MS (LC-MS/MS).

The most commonly employed approach to study protein–protein interactions *in vivo* is affinity purification-MS (AP-MS; Gingras et al., 2007; Vermeulen et al., 2008; Meyer and Selbach, 2015). In AP-MS workflows, a protein of interest (bait protein) is co-purified with its interaction partners and the purified proteins are subsequently identified by LC-MS/MS. Purification of the bait protein can be achieved using antibodies that specifically bind to the endogenous bait protein. Alternatively, epitope tags can be employed that enable robust and reproducible purification of the bait protein and its interaction partners using highly specific affinity matrices. The latter approach is especially beneficial when antibodies recognizing the bait protein are not available; however, the introduction of epitope tags usually involves overexpression of the bait protein and can lead to non-physiological interactions.

The power of AP-MS for high-throughput discovery of protein–protein interactions has been exemplified by recent landmark studies from the Mann and Gygi laboratories that demonstrated systematic analyses of human protein–protein interactions and mapped 28,500 and 23,744 unique interactions, respectively (Hein et al., 2015; Huttlin et al., 2015). These studies represent a milestone in the long-term effort to comprehensively map human protein–protein interactions.

In addition to AP-MS, co-fractionation strategies have been employed to study cellular organelles and protein complexes. The Mann laboratory has employed biochemical fractionation based on density gradient centrifugation to define the composition of cellular organelles (Andersen et al., 2003; Foster et al., 2006). More recently, Havugimana et al. (2012) and Wan et al. (2015) employed extensive biochemical fractionation and MS to determine the composition of soluble protein complexes in human cells and in cells from diverse metazoan model organisms.

## Resolving Transient Protein–Protein Interactions

Most studies conducted have so far investigated steady state protein–protein interactions, leaving the temporal and spatial aspects of protein–protein interactions largely disregarded. Mapping transient protein–protein interactions during cellular signaling and in response to cellular perturbations remains a major future challenge. For instance, changes in protein interactions induced by growth factor stimulation or cellular stress, as well as interactions between PTM-catalyzing enzymes and substrates, can often not be captured using conventional methods for analyses of protein interactions. Accordingly, efforts are ongoing to design proteomics methods that permit analysis of transient and low affinity protein interactions.

### AP-MS Combined with Quantitative Mass Spectrometry-Based Proteomics

**FIGURE 2:**
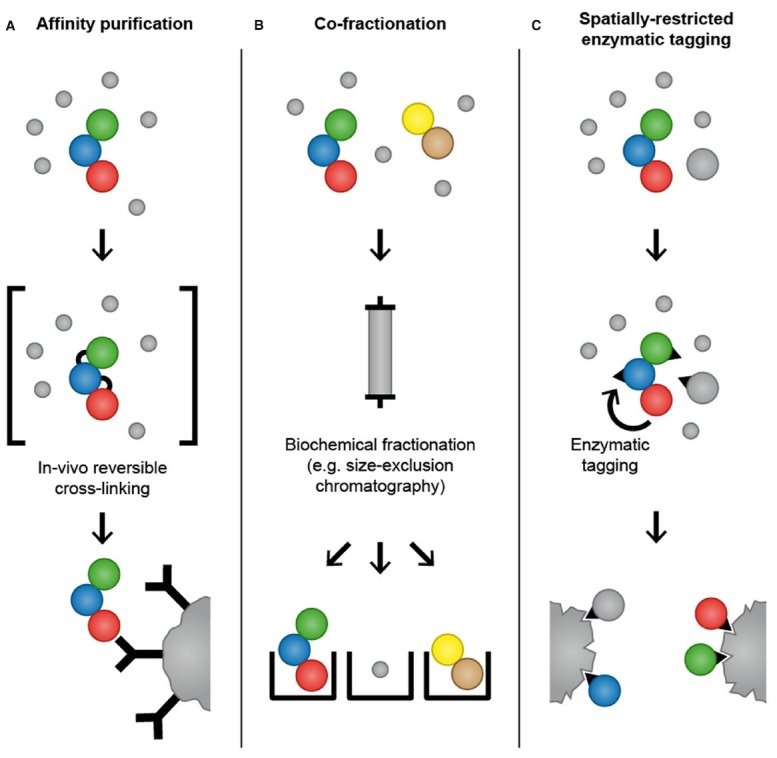
**Mass spectrometry-based proteomics methods for analysis of temporal and spatial aspects of protein–protein interactions.** In affinity purification approaches, an antibody that specifically binds to endogenously expressed bait protein is used to purify the protein of interest and its interaction partners. Alternatively, a bait protein fused to an epitope tag is ectopically expressed in cells and purified using affinity matrices or tag-specific antibodies. To increase the probability of capturing transient and weak interactions, chemicals that mediate protein–protein crosslinks can be applied to cells before lysis to “freeze” interactions by forming reversible covalent bonds between adjacent amino acids **(A)**. In co-fractionation-based methods, proteins are subjected to extensive fractionation, for instance by high-performance size-exclusion chromatography, and the precise co-elution of two proteins is used as evidence for their interaction **(B)**. In spatially restricted enzymatic tagging BirA* or APEX is fused to a protein of interest and ectopically expressed in cells. Biotinylation of proximal proteins is triggered by the addition of biotin for 24 h (BioID) or biotin-phenol for 1 min (APEX). Cells are lysed under denaturing conditions and biotinylated proteins are recovered using streptavidin followed by LC-MS/MS analysis **(C)**.

Affinity purification combined with quantitative MS-based proteomics can be used to identify dynamic protein–protein interactions (Figure 2). In this approach, affinity purifications are performed under different conditions and the relative abundance of interaction partners is then determined by quantitative MS-based approaches, including metabolic and chemical labeling as well as label-free methods (Ong and Mann, 2005; Bantscheff et al., 2012). Affinity purification is often combined with stable isotope labeling with amino acids in cell culture (SILAC) to monitor protein interactomes after different types of cellular perturbations, including DNA damage (Mosbech et al., 2012; Brown et al., 2015) and ligand stimulation (Satpathy et al., 2015). In addition, this approach has been applied to study the temporal dynamics of protein interactions during cell cycle progression (Hubner et al., 2010; Pagliuca et al., 2011).

Recently, data-independent acquisition (DIA) methods have been employed to map changes in protein–protein interactions after cellular perturbations. Analysis of peptide samples from affinity purification experiments has typically been performed using data-dependent acquisition methods (DDA). Due to the semi-stochastic precursor ion selection in DDA methods, the complete set of peptides can often not be reproducibly identified across all samples. In DIA methods, fragment spectra for the entire mass range are acquired by co-isolating precursor ions in isolation windows of selected m/z ranges. Collins et al. (2013) have described a method for mapping dynamic changes in protein–protein interactions by combining affinity purification with DIA using MS-sequential window acquisition of all theoretical spectra (MS-SWATH). The authors have analyzed interaction partners of 14-3-3β in cells stimulated with insulin-like growth factor for different time periods and reproducibly quantified 1,967 proteins across all samples. A similar approach has been used by Lambert et al. (2013) to map the interaction partners of wild type and mutant forms of CDK4 as well as to probe the effects of Hsp90 inhibition on CDK4 interactions.

### *In Vivo* Reversible Crosslinking

A complementary approach to affinity purification that aims to capture transient and low affinity protein–protein interactions is reversible chemical crosslinking (Hall and Struhl, 2002; Vasilescu et al., 2004; Klockenbusch and Kast, 2010; Smith et al., 2011). Chemicals that mediate protein crosslinks, such as formaldehyde, are applied to cells before lysis to “freeze” protein–protein interactions *in vivo* by forming reversible covalent bonds between adjacent amino acids, thereby providing a snapshot of the protein interactome (Figure 2). Following crosslinking, cells are lysed and proteins are subjected to conventional affinity purification protocols. Crosslinks are reversed after purification, often simply by boiling, and affinity-purified proteins are identified by LC-MS/MS. In addition to formaldehyde, other crosslinkers that are commonly used for protein–protein interaction studies are NHS-esters and imidates that react with primary amines in the proteins to yield stable amide bonds. If crosslinking is combined with epitope tagging of the bait protein and purification with affinity matrices such as GFP-Trap and Ni-NTA, cell lysis and washing can be performed under denaturing conditions, thus enabling the recovery of poorly soluble proteins and reducing contamination with non-physiological interactions that might occur during the purification (Tagwerker et al., 2006). Formaldehyde-based crosslinking and purification under denaturing conditions has been employed to identify interaction partners of Skp1, an essential component of the SCF ubiquitin ligase complex, and to map the dynamic interaction partners of the 26S proteasome across cell cycle phases (Tagwerker et al., 2006; Kaake et al., 2010). The fact that the crosslinking procedure requires optimization for different cell types and bait proteins might be the reason that this technique has not so far been frequently used for the investigation of transient protein–protein interactions.

### Co-fractionation Combined with Quantitative Mass Spectrometry

Kristensen et al. (2012) have developed a method that employs quantitative MS based on SILAC and high-performance size-exclusion chromatography to monitor changes in the cellular interactome in response to growth factor stimulation (Figure 2). Using this approach, the authors have identified 350 proteins whose association with a complex increased or decreased after cells were stimulated with the epidermal growth factor. A particular feature of this method is that it allows mapping of dynamic changes in the cellular interactome without the need to overexpress bait proteins and perform affinity purifications. In addition, size-exclusion chromatography enables the heterogeneity of protein complexes within the cells to be determined, by monitoring the distribution of a protein among different complexes. Another advantage of this method is that it provides the possibility to analyze the interactome within a single subcellular compartment, thereby providing a spatial dimension and avoiding the risk of non-physiological interactions that can occur after cell lysis and loss of cellular compartmentalization.

## Resolving Spatial Organization of Protein–Protein Interactions by Proximity Tagging

In addition to defining transient protein–protein interactions, another challenge lies in resolving the spatial organization of protein interaction networks. In affinity purification approaches, proteins localized to different cellular compartments are mixed during cell lysis and subjected to purification under native conditions, which might lead to the formation of non-physiological interactions. Recently developed methods for spatially restricted enzymatic tagging using the promiscuous biotin ligase BirA* (BioID) or the engineered ascorbate peroxidase (APEX) can be employed to overcome this problem and preserve the spatial dimension of interactions (Roux et al., 2012; Rhee et al., 2013).

### Biotin Ligase-Based Proximity Tagging (BioID)

BirA is a biotin ligase from *E. coli* that activates biotin to biotinoyl 5-AMP (bioAMP) in an ATP-dependent reaction (Chapman-Smith and Cronan, 1999). Biotinoyl 5′-AMP is then transferred to substrate proteins containing a specific BirA recognition sequence (Beckett et al., 1999). An engineered mutant form of BirA (R118G) with abolished substrate specificity and reduced affinity for biotinoyl 5′-AMP promiscuously biotinylates proteins in its proximity (Choi-Rhee et al., 2004; Cronan, 2005). Roux et al. (2012) devised a method called BioID in which the promiscuous biotin ligase BirA* is fused to a protein of interest and expressed in mammalian cells. After incubation of the cells with biotin, the BirA*-fusion protein biotinylates proteins in its proximity (Figure 2). Subsequently, cells are lysed under denaturing conditions and biotinylated proteins are selectively isolated using streptavidin and identified by LC-MS/MS. The authors tested the utility of BioID by fusing BirA* to the nuclear envelope (NE) component lamin A (LaA) that is highly insoluble and therefore difficult to study with conventional methods for interactome analysis. Analysis of biotinylated proteins in cells expressing BirA*-LaA by LC-MS/MS identified known LaA interactors as well as the novel NE component SLAP75 (Roux et al., 2012). BioID possesses several advantages over conventional affinity purification. Firstly, BirA*-based biotinylation of proteins occurs in living cells and therefore non-physiological interactions that might occur after cell lysis and loss of cellular compartmentalization are avoided. Secondly, proximity-dependent biotinylation by the promiscuous biotin ligase BirA* can capture low affinity interactions that will frequently be lost in conventional affinity purification. Furthermore, BioID allows the use of denaturing lysis conditions, which helps to identify proteins that are insoluble under commonly used native lysis conditions and reduces contamination with non-specific binders. However, BioID also has limitations that should be considered during experimental design. Activated biotin targets primary amines (predominantly lysine residues) and the efficacy of the biotinylation depends on the number and availability of primary amines in proteins (Roux et al., 2013). As result, the abundance of the purified biotinylated proteins does not necessarily correlate with the strength or stoichiometry of the association. Moreover, biotinoyl 5′-AMP has a half-life of minutes, which might lead to a large labeling radius (Rhee et al., 2013). In the BioID-LaA experiment, the authors showed that histone proteins constitute only a small fraction of the identified proteins, although they are lysine rich and highly abundant in the nucleus, which provides evidence against the idea that BioID generates widespread biotinylation (Roux et al., 2012). Importantly, BioID does not distinguish interaction from proximity, which needs to be taken into account during data analysis. BioID has been successfully employed to identify interaction partners of proteins and to characterize the composition of subcellular organelles, such as the centrosomes and the nuclear pore, which are otherwise refractory to traditional approaches (Couzens et al., 2013; Firat-Karalar et al., 2014; Coyaud et al., 2015; Dingar et al., 2015; Rodriguez-Fraticelli et al., 2015; Zhou et al., 2015). A recent study employed BioID to identify over 50 putative substrates of the ubiquitin ligase SCF^β-TrCP1/2^ indicating a potential application of BioID for the analysis of substrates of PTM-catalyzing enzymes (Coyaud et al., 2015). The Gingras laboratory has performed a side-by-side comparison of AP-MS and BioID for analyzing interaction partners of chromatin-associated proteins (Lambert et al., 2015). Interestingly, they concluded that BioID enables the identification of a larger number of interaction partners and that identified interaction partners are significantly less abundant than interaction partners identified by AP-MS. Another observation from this study is the relatively small overlap between the interaction partners identified by AP-MS and BioID, suggesting that both approaches have a bias for specific subsets of proteins and might have a complementary value for comprehensive identification of protein interaction partners.

### Ascorbate Peroxidase-Based Proximity Tagging

Another enzymatic proximity tagging approach developed by the Ting laboratory uses an engineered ascorbate peroxidase (APEX) (Martell et al., 2012). APEX is a monomeric mutant derived from the plant APEX with increased enzymatic activity. Like wild type peroxidase, APEX catalyzes H_2_O_2_-dependent polymerization and local deposition of DAB (3,3′-diaminobenzidine), which subsequently recruits electron dense osmium, yielding electron microscopy (EM) contrast (Lam et al., 2015). Based on the observation that APEX is active in all cellular compartments and withstands strong EM fixation, Martell et al. (2012) demonstrated the utility of APEX for EM analysis of a variety of mammalian organelles and specific proteins.

In addition to DAB, APEX also oxidizes numerous phenol derivatives such as biotin-phenol to phenoxyl radicals that covalently react with electron-rich amino acids. In cells expressing APEX fused to a protein of interest, biotinylation of proximal proteins is initiated by incubating cells with biotin-phenol and H_2_O_2_ for 1 min. The proximal proteins can subsequently be purified using streptavidin under denaturing conditions and identified by LC-MS/MS analysis (Figure 2). Rhee et al. (2013) selected mitochondria as a model organelle for testing APEX-based identification of organelle proteins. To test the spatially restricted labeling capacity of APEX, mitochondrial matrix-targeted APEX was used to investigate the protein composition of the mitochondrial matrix and inner mitochondrial membrane. Using LC-MS/MS, the authors have identified 495 proteins, 94% of which had prior mitochondrial annotation. Thirty-one of those 495 proteins had never been correlated with mitochondria and are therefore potentially novel mitochondrial proteins. Of note, only subunits with exposure to matrix space were identified, indicating that phenoxyl radicals do not pass through the inner mitochondrial membrane, proving further the specificity of APEX-based proximity tagging (Lam et al., 2015).

APEX-based proximity tagging can provide spatially and temporally resolved proteomic maps and can be potentially employed to study weak and dynamic protein interactions as well as enzyme-substrate relations. APEX requires only 1 min to label proximal proteins rather than the 24 h required for the BioID method. It therefore, has a better temporal resolution and offers a better platform to study transient protein–protein interactions under different conditions and time points. Furthermore, phenoxyl radicals are short lived (<1 ms) and therefore have a small labeling radius (<20 nm). It is worth mentioning that APEX can also be used to confirm the subcellular localization of target proteins using EM or fluorescent microscopy. To date, the applicability of APEX beyond the mapping of proteins in membrane-bound cellular organelles has not been demonstrated, and it remains to be addressed if APEX-based proximity tagging is suitable for analysis of interaction partners of individual proteins or protein substrates of PTM-catalyzing enzymes.

## Conclusion

Mass spectrometry-based proteomics has delivered unprecedented insights into human protein interaction networks. To date, most studies have focused on mapping steady-state protein–protein interactions. Future challenges remain in the identification of transient and low affinity interactions during cellular signaling, as well as in understanding the spatial organization of protein interaction networks. Although affinity purification combined with quantitative MS-based proteomics is a powerful approach for the identification of dynamic protein interactions, transient and low affinity interactions, such as those induced by growth factor stimulation or cellular stress, are frequently lost. *In vivo* chemical crosslinking, in which chemicals that form reversible covalent bonds are applied to cells before lysis to “freeze” protein–protein interactions can help to identify these interactions. The need to optimize the crosslinking procedure for different cell types and bait proteins hinders the routine use of this method for analyzing transient protein interactions. In addition to AP-MS, approaches based on protein co-fractionation combined with quantitative MS have been successfully employed to analyze transient protein interactions during cellular signaling. Spatially restricted enzymatic tagging approaches, such as BioID and APEX, preserve the spatial organization of protein interaction networks and enable analysis of protein interactions in insoluble structures, thereby complementing AP-MS. Importantly, these approaches do not enable a distinction to be made between interaction partners and non-interacting proximal proteins. Therefore, combining affinity purification and spatially restricted enzymatic tagging could help to produce a more accurate and comprehensive picture of protein–protein interaction networks of interest. This strategy has the potential to become a standard procedure for protein interaction studies, as has already been exemplified by a recent study that focused on chromatin-associated protein complexes (Lambert et al., 2015)

## Author Contributions

JY, SW, and PB prepared and wrote the manuscript.

### Conflict of Interest Statement

The authors declare that the research was conducted in the absence of any commercial or financial relationships that could be construed as a potential conflict of interest.
